# Vital Signs: Avoidable Deaths from Heart Disease, Stroke, and Hypertensive Disease — United States, 2001–2010

**Published:** 2013-09-06

## Abstract

**Background:**

Deaths attributed to lack of preventive health care or timely and effective medical care can be considered avoidable. In this report, avoidable causes of death are either preventable, as in preventing cardiovascular events by addressing risk factors, or treatable, as in treating conditions once they have occurred. Although various definitions for avoidable deaths exist, studies have consistently demonstrated high rates in the United States. Cardiovascular disease is the leading cause of U.S. deaths (approximately 800,000 per year) and many of them (e.g., heart disease, stroke, and hypertensive deaths among persons aged <75 years) are potentially avoidable.

**Methods:**

National Vital Statistics System mortality data for the period 2001–2010 were analyzed. Avoidable deaths were defined as those resulting from an underlying cause of heart disease (ischemic or chronic rheumatic), stroke, or hypertensive disease in decedents aged <75 years. Rates and trends by age, sex, race/ethnicity, and place were calculated.

**Results:**

In 2010, an estimated 200,070 avoidable deaths from heart disease, stroke, and hypertensive disease occurred in the United States, 56% of which occurred among persons aged <65 years. The overall age-standardized death rate was 60.7 per 100,000. Rates were highest in the 65–74 years age group, among males, among non-Hispanic blacks, and in the South. During 2001–2010, the overall rate declined 29%, and rates of decline varied by age.

**Conclusions:**

Nearly one fourth of all cardiovascular disease deaths are avoidable. These deaths disproportionately occurred among non-Hispanic blacks and residents of the South. Persons aged <65 years had lower rates than those aged 65–74 years but still accounted for a considerable share of avoidable deaths and demonstrated less improvement.

**Implications for Public Health Practice:**

National, state, and local initiatives aimed at improving health-care systems and supporting healthy behaviors are essential to reducing avoidable heart disease, stroke, and hypertensive disease deaths. Strategies include promoting the ABCS (aspirin when appropriate, blood pressure control, cholesterol management, and smoking cessation), reducing sodium consumption, and creating healthy environments.

## Introduction

In the 1970s, a method for measuring the quality of medical care through identifying “untimely and unnecessary” deaths was proposed (1). This concept has since been expanded to include deaths attributed to lack of preventive health care (i.e., preventing cardiovascular events by addressing risk factors) or timely and effective medical care (i.e., treating patients who have cardiovascular conditions); these deaths are defined as avoidable (2). Although no standard method for measuring avoidable deaths exists, Canada (3), the United Kingdom (4), and the European Union (5) have introduced avoidable death measures for their surveillance systems. In several previous studies, the United States ranked higher in avoidable death rates compared with other industrialized countries (6).

Heart disease is the leading cause of death in the United States, and cardiovascular disease accounts for nearly 30% of all deaths annually (nearly 800,000 deaths) (7). Many heart disease and stroke deaths could be avoided through improvements in lifestyle behaviors, treatment of risk factors, and addressing the social determinants of health (i.e., economic and social conditions that influence the health of individuals and communities). Unhealthy lifestyle behaviors (e.g., tobacco use, inadequate physical activity, poor diet, and excessive alcohol use) coupled with uncontrolled hypertension, elevated cholesterol, and obesity account for 80% of ischemic heart disease mortality and approximately 50% of stroke mortality in high-income countries such as the United States (8). Hypertension is the single most important risk factor for stroke, and its control is essential to reducing death from stroke (8). Additional medical interventions, such as secondary prevention and evidence-based procedures to treat ischemic heart disease and stroke, have been shown to reduce deaths in the United States (9,10).

This report describes the epidemiology of avoidable deaths from heart disease, stroke, and hypertensive disease in the United States, presents trends in avoidable death rates for these causes, and documents geographic disparities by state and county.

## Methods

Mortality data from the National Vital Statistics System for the period 2001–2010 were analyzed. Bridged-race July 1 population estimates produced by the U.S. Census Bureau in collaboration with the National Center for Health Statistics were compiled using intercensal estimates for the period 2001–2009 and postcensal estimates for 2010.

In this report, avoidable deaths include all deaths among persons aged <75 years with an underlying cause of ischemic heart disease (*International Classification of Diseases, 10th Revision* [ICD-10] codes I20–I25), cerebrovascular disease (stroke) (I60–I69), hypertensive disease (I10–I15), or chronic rheumatic heart disease (I05–I09) (2). The analyses were limited to persons aged <75 years because the life expectancy of the total U.S. population in 2010 was 78.7, and 100% of these deaths in persons aged <75 years were considered to be preventable in accordance with previous analyses (3–5). Age-standardized death rates were calculated by sex, race/ethnicity,* and the decedent’s state of residence at time of death, and trends were analyzed for the period 2001–2010 using joinpoint regression to calculate the average annual percentage change (AAPC). Rate comparisons were made using rate ratios (RRs). County-level rates for combined years 2008–2010 were calculated using a spatial empirical Bayesian smoothing technique to enhance the stability of the rates (11).

## Results

In 2010, the total number of avoidable deaths from heart disease, stroke, and hypertensive disease was 200,070, and the death rate was 60.7 per 100,000 population (Table 1). Death rates in 2010 were highest in the oldest age group (65–74 years) (401.5 per 100,000) and lowest in the youngest age group (0–34 years) (1.9 per 100,000); however, 56% of the deaths (n = 112,329) occurred among those aged <65 years. Avoidable deaths were higher among males (83.7 per 100,000) than females (39.6) and blacks (107.3) compared with other races/ethnicities. Rates for blacks and American Indians/Alaska Natives were statistically significantly higher than those for whites (RR = 1.9 and 1.2, respectively), whereas rates for Hispanics and Asian/Pacific Islanders were significantly lower (RR = 0.8 and 0.6, respectively).

**FIGURE 1:**
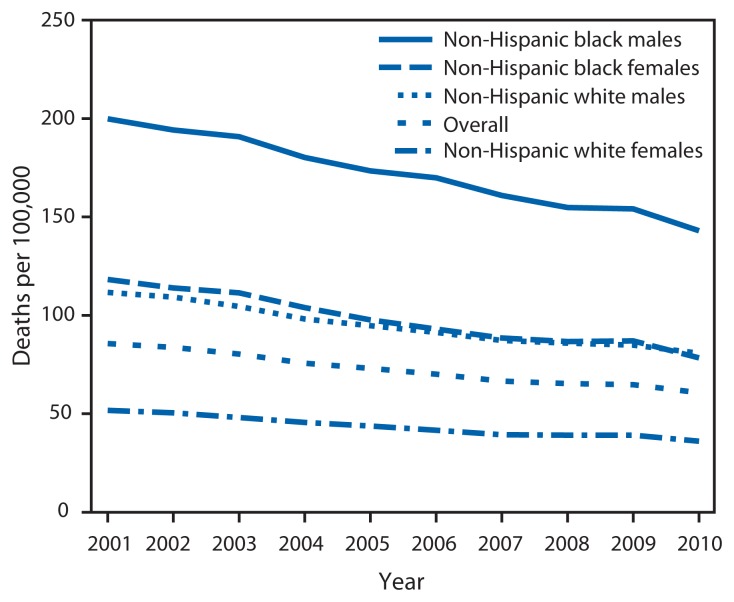
Age-adjusted rates^*^ of avoidable death from heart disease, stroke, and hypertensive disease^†^ among non-Hispanic blacks and non-Hispanic whites, by sex — United States, 2001–2010 ^*^Rates are age-standardized to the U.S. standard 2000 population. ^†^Avoidable deaths from heart disease, stroke, and hypertensive disease are defined as all deaths occurring in persons aged <75 years with an underlying cause of ischemic heart disease, cerebrovascular disease, hypertensive disease, or chronic rheumatic heart disease.

From 2001 to 2010, the avoidable death rate from heart disease, stroke, and hypertensive disease decreased 29%. The AAPC shows that rates decreased sharply for the 65–74 years age group (AAPC = −5.1), declined more gradually in the 55–64 years age group (AAPC = −3.3), declined minimally in the 35–54 years age group (AAPC = −0.8), and did not change in the youngest age group (Table 1). Declines occurred among both sexes and all race/ethnicity groups. Temporal trends for blacks and whites from 2001 to 2010 showed a decrease over time for all groups; however, black males consistently experienced the highest avoidable death rates throughout the period, and black females showed rates similar to white males (Figure 1).

**FIGURE 2:**
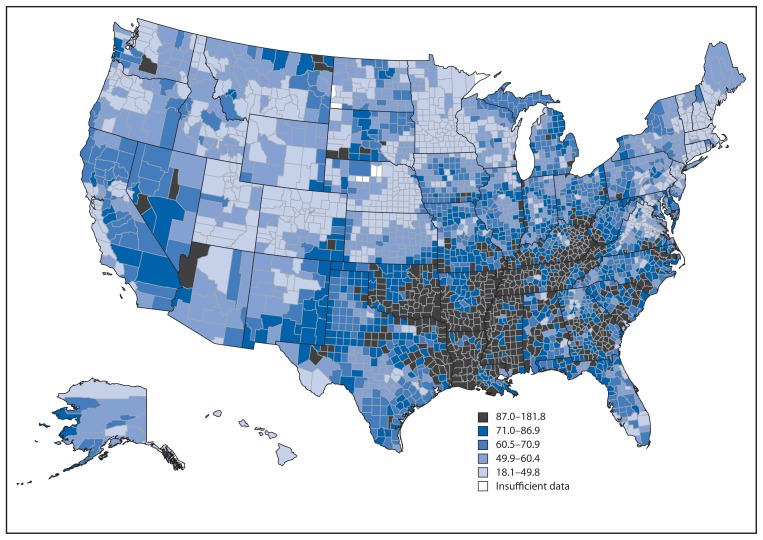
Rates^*^ of avoidable death from heart disease, stroke, and hypertensive disease,^†^ by county — United States, 2008–2010^§^ ^*^ Per 100,000 population. Rates are averaged over the 2008–2010 period and age-standardized to the U.S. standard 2000 population. Rates are spatially smoothed to enhance the stability of rates in counties with small populations. ^†^Avoidable deaths from heart disease, stroke, and hypertensive disease are defined as all deaths occurring in persons aged <75 years with an underlying cause of ischemic heart disease, cerebrovascular disease, hypertensive disease, or chronic rheumatic heart disease. ^§^Additional maps by race/ethnicity and sex are available on the Interactive Atlas for Heart Disease and Stroke at http://nccd.cdc.gov/dhdspatlas.

By state, avoidable deaths from heart disease, stroke, and hypertensive disease in 2010 ranged from 36.3 to 99.6 per 100,000 population in Minnesota and the District of Columbia, respectively, a greater than two-fold difference (Table 2). All states experienced declines in rates for these avoidable causes during 2001–2010, ranging from an AAPC of −1.6 in Wyoming to an AAPC of −6.1 in New Hampshire. By county, the highest avoidable death rates in combined years 2008 to 2010 were concentrated primarily in the southern Appalachian region and much of Tennessee, Arkansas, Mississippi, Louisiana, and Oklahoma, whereas the lowest rates were located in the West, Midwest, and Northeast census regions† (Figure 2). Within states, substantial variation often occurred in the county rates, with some states experiencing a fourfold difference in death rates among counties (e.g., Colorado, Virginia, Kentucky, and Maryland).

## Conclusions and Comment

Avoidable death rates from heart disease, stroke, and hypertensive disease in the United States vary by age, race/ethnicity, sex, place, and time. In 2010, an estimated 200,070 avoidable deaths from these causes occurred in the United States. Although the highest death rate occurred among those aged 65–74 years, the younger age groups (aged <65 years) still experienced a substantial number of avoidable deaths and a relatively slower rate of decline during 2001–2010. The avoidable death rate among blacks was nearly twice that of whites. Counties with the highest avoidable death rates were located primarily in the South census region.

The overall decrease in deaths from ischemic heart disease (the largest contributing cause of the avoidable deaths measured) can be attributed to both improvements in risk factors and changes in cardiac treatments (9). The variation in age-specific rates of decline for avoidable deaths from heart disease, stroke, and hypertensive disease, with slower declines in the younger age group, could have resulted from multiple factors. Differential temporal trends in the percentage of adults without health insurance by age group are one possibility. Whereas the percentage of adults aged 18–64 years with no health insurance increased from 17% in 2001 to 22% in 2010, it remained at ≤2% among adults aged ≥65 years (because of Medicare coverage in this population) (12). Although avoidable death rates in those aged ≥35 years have declined over this interval, the increase in percentage without insurance among the younger age groups might have limited their access to preventive screenings and early treatment of high blood pressure and elevated cholesterol and, therefore, contributed to their slower decline in rates (13,14). Age-specific differences in risk factor management also might have contributed to the slower decline in the younger age group. Compared with persons aged ≥60 years, during 2009–2010, adults aged 18–39 years with high blood pressure experienced lower rates of treatment (43.5% versus 83.6%) and control (28.6% versus 47.0%) and saw no improvements in those rates from 2001 to 2010 (15). Furthermore, among persons aged 35–44 years, stroke hospitalizations increased during 2001–2006, whereas they remained constant for those aged 45–54 years and decreased among those aged 55–64 years (16). The finding of a slower decline in avoidable deaths in younger age groups in this report highlights the importance of improving prevention, diagnosis, and treatment efforts in younger adults.

Blacks experienced a disproportionate number of avoidable deaths from heart disease, stroke, and hypertensive disease, with nearly twice the rate as whites. Risk for avoidable death is particularly high among black males; in 2010, their rate was approximately 80% higher than that of white males and black females. Compared with whites, blacks have higher prevalence of cardiovascular disease risk factors, including high blood pressure, diabetes, obesity, physical inactivity, low fruit and vegetable consumption, and poor low-density lipoprotein cholesterol control (13). In addition, previous studies suggest that the U.S. black-white disparity in avoidable mortality reflects differences in education, income, living conditions, and access to health care (2). Interventions aimed at addressing these social determinants of health in combination with effective treatment and control of risk factors could help reduce black-white disparities in avoidable deaths (17).

State-level and county-level differences in avoidable death rates from heart disease, stroke, and hypertensive disease suggest the need for interventions that target areas with the highest rates and work with the resources, policies, and programs already existing in those areas. In 2010, the states with the highest avoidable death rates were located primarily in the South (e.g., District of Columbia, Mississippi, Oklahoma, Tennessee, and Louisiana). The states with the lowest rates were Minnesota, Utah, Colorado, Connecticut, and New Hampshire. During 2001–2010, all states experienced declines in avoidable death rates; however, some of the states that already had the lowest rates saw some of the steepest declines in absolute percentage change and AAPC (e.g., New Hampshire and Rhode Island), whereas some states with the highest rates had the slowest declines (e.g., Oklahoma and Arkansas). Moreover, variation in avoidable deaths exists within states by county (Figure 2). These geographic disparities support the need for local-level policy changes and system-level changes (e.g., promoting community design that increases access to sidewalks and bike lanes, improving the local food environment, enhancing worksite wellness programs, and improving insurance coverage) to improve access to quality health care and enhance or create the physical, social, and built environments needed to support healthy lifestyles (18).

The findings in this report are subject to at least four limitations. First, ICD-10 codes might misclassify cause of death, especially for stroke; however, more classification issues typically are experienced among the very old, a population not included in this study (19). Second, race and ethnicity might not be reported accurately on death certificates; this typically leads to underreporting of American Indian/Alaska Native, Asian/Pacific Islander, and Hispanic race/ethnicity (20). Third, death rate data in this report are based on residency at time of death and not on the state in which a person spent the majority of his or her life. Finally, there is no universally agreed upon definition for avoidable heart disease and stroke deaths, which could limit ability to compare these results with other studies. The strength of the methodology used in this report (2) is that it focuses on both preventable and treatable conditions whereas other methodologies might focus on one or the other. Other definitions of avoidable deaths resulting from these causes could lead to differing estimates, but most likely similar trends and associations.

Strong collaboration between health care and public health is critical to reduce the burden of avoidable deaths from heart disease, stroke, and hypertensive disease. The Million Hearts initiative is a national effort working to improve access and quality of care to reduce the incidence of heart disease and stroke through community and clinical prevention strategies. These strategies include promoting the ABCS of heart health (aspirin when appropriate, blood pressure control, cholesterol management, and smoking cessation); use of health information technology (to help doctors track and treat patients with high blood pressure and elevated cholesterol); and team-based care (an evidence-based collaborative model that is more effective in controlling high blood pressure and cholesterol than a single health-care provider working alone), as well as community prevention strategies, including tobacco control and reducing sodium and eliminating trans fats from foods. In addition, state-level and local-level initiatives are working to enhance community and clinical collaborations. For example, the state of Massachusetts is developing an electronic referral system and data exchange to enhance communication between clinicians and community resources such as telephone quitlines for smokers, physical activity supports, and blood pressure self-management to prevent heart disease and stroke risk factors more effectively. The Sodium Reduction in Communities Program is a county-level effort to help reduce sodium in schools, restaurants, and other venues while also educating the public on sodium reduction (21). Reducing sodium in foods can aid in control of high blood pressure. Finally, individuals can work toward reducing their own heart disease and stroke risk. The American Heart Association has defined seven simple steps to a healthier heart to help individuals increase healthy behaviors (22). Although this report defined avoidable deaths as those occurring in persons aged <75 years based on life expectancy in the United States, these public health, health-care, community, and patient strategies can help reduce deaths from heart disease and stroke in the United States across all age groups.

## Reported by

*Linda J. Schieb, MSPH, Sophia A. Greer, MPH, Matthew D. Ritchey, DPT, Mary G. George, MD, Michele L. Casper, PhD, Div for Heart Disease and Stroke Prevention, National Center for Chronic Disease Prevention and Health Promotion, CDC.* ***Corresponding contributor:*** *Linda J. Schieb, lschieb@cdc.gov, 770-488-5348.*

**Key Points**

- Minimal declines in avoidable deaths from heart disease, stroke, and hypertensive disease occurred in younger age groups (0–34 and 35–54 years) compared with older age groups (55–64 and 65–74 years).
- Non-Hispanic blacks experience a disproportionately large number of avoidable deaths, with nearly twice the rate of avoidable death as non-Hispanic whites.
- Rates of avoidable deaths from heart disease, stroke, and hypertensive disease are highest in the South.
- Additional information is available at http://www.cdc.gov/vitalsigns.

## Figures and Tables

**TABLE 1:** Number and rate of avoidable deaths* from heart disease, stroke, and hypertensive disease, by age, sex, and race/ethnicity — United States, 2001 and 2010

Characteristic	2001			2010				Average annual % change 2001 to 2010
	
	No.	Rate†	(95% CI)	No.	Rate†	(95% CI)	Rate ratio	
**Total**	**227,961**	**85.7**	**(85.4–86.1)**	**200,070**	**60.7**	**(60.4–61.0)**	**—**	−**3.8**§
**Age group (yrs)**								
0–34	2,858	2.0	(2.0–2.1)	2,765	1.9	(1.8–2.0)	Referent	0.4¶
35–54	46,426	55.0	(54.5–55.5)	43,884	51.0	(50.6–51.5)	26.8§	−0.8§
55–64	61,015	243.0	(241.1–245.0)	65,680	178.6	(177.2–180.0)	94.0§	−3.3§
65–74	117,662	640.0	(636.4–643.7)	87,741	401.5	(398.8–404.1)	211.3§	−5.1§
**Sex**								
Males	146,189	116.9	(116.3–117.5)	132,215	83.7	(83.2–84.2)	Referent	−3.7§
Females	81,772	57.9	(57.5–58.3)	67,855	39.6	(39.3–39.9)	0.5§	−4.1§
**Race/Ethnicity and sex**								
White, non-Hispanic	168,732	80.4	(80.0–80.7)	142,448	57.8	(57.5–58.1)	Referent	−3.6§
Males	111,265	111.7	(111.0–112.4)	96,451	80.9	(80.3–81.4)	—	−3.5§
Females	57,467	51.7	(51.3–52.2)	45,997	36.1	(35.7–36.4)	—	−3.9§
Black, non-Hispanic	40,398	154.0	(152.5–155.6)	37,348	107.3	(106.2–108.5)	1.9§	−3.9§
Males	23,050	199.8	(197.2–202.5)	22,417	143.0	(141.1–144.9)	—	−3.6§
Females	17,348	118.3	(116.5–120.0)	14,931	78.4	(77.2–79.7)	—	−4.4§
Hispanic**	12,884	68.2	(67.0–69.4)	13,855	45.4	(44.7–46.2)	0.8§	−4.5§
Males	8,205	93.0	(91.0–95.1)	9,175	63.2	(61.8–64.5)	—	−4.3§
Females	4,679	46.8	(45.4–48.1)	4,680	29.7	(28.8–30.6)	—	−5.1§
AI/AN, non-Hispanic**	1,368	86.9	(82.1–91.6)	1,498	66.9	(63.5–70.4)	1.2§	−3.0§
Males	851	113.5	(105.7–121.4)	965	90.0	(84.2–95.8)	—	−2.5§
Females	517	63.1	(57.6–68.6)	533	45.9	(42.0–49.9)	—	−3.8§
Asian/Pacific Islander, non-Hispanic**	4,579	50.5	(49.0–52.0)	4,921	33.6	(32.6–34.5)	0.6§	−4.3§
Males	2,818	67.9	(65.3–70.4)	3,207	47.3	(45.7–49.0)	—	−3.7§
Females	1,761	36.0	(34.3–37.7)	1,714	21.9	(20.9–23.0)	—	−5.4§

**Abbreviations:** CI = confidence interval; AI/AN = American Indian/Alaska Native.

* Avoidable deaths from heart disease, stroke, and hypertensive disease are defined as all deaths occurring in persons aged <75 years with an underlying cause of ischemic heart disease, cerebrovascular disease, hypertensive disease, or chronic rheumatic heart disease.

† Per 100,000 population. Rates are age-standardized to the U.S. standard 2000 population except for age-specific rates.

§ Statistically different from zero at alpha = 0.05.

¶ Results based on small numbers.

** Numbers and rates for AI/ANs, Hispanics, and Asians/Pacific Islanders might be underreported because of coding issues on death certificates.

**TABLE 2:** Number and rate of avoidable deaths* from heart disease, stroke, and hypertensive disease, by state — United States, 2001 and 2010

State	2001			2010			Average annual % change 2001 to 2010§
	
	No.	Rate†	(95% CI)	No.	Rate†	(95% CI)	
Alabama	4,290	96.2	(93.3–99.1)	3,998	75.2	(72.8–77.5)	−2.5
Alaska	313	69.8	(61.7–77.9)	359	52.5	(46.9–58.1)	−2.6
Arizona	3,798	75.2	(72.8–77.5)	3,686	52.5	(50.8–54.2)	−3.8
Arkansas	2,910	106.5	(102.6–110.4)	2,849	87.5	(84.2–90.7)	−1.9
California	22,673	79.6	(78.5–80.6)	19,734	54.4	(53.6–55.1)	−4.3
Colorado	2,070	57.0	(54.5–59.5)	2,041	39.9	(38.1–41.7)	−3.9
Connecticut	2,203	66.0	(63.3–68.8)	1,651	41.8	(39.8–43.8)	−5.7
Delaware	682	85.9	(79.4–92.3)	613	59.8	(55.0–64.6)	−3.6
District of Columbia	718	137.8	(127.7–147.9)	580	99.6	(91.4–107.8)	−3.7
Florida	15,317	82.8	(81.5–84.1)	13,143	57.3	(56.3–58.3)	−4.1
Georgia	6,569	93.8	(91.6–96.1)	6,480	66.7	(65.1–68.4)	−4.0
Hawaii	769	63.9	(59.4–68.5)	666	44.1	(40.7–47.5)	−3.6
Idaho	775	67.4	(62.6–72.1)	790	49.0	(45.6–52.5)	−4.2
Illinois	10,096	89.9	(88.2–91.7)	8,182	61.9	(60.6–63.3)	−4.1
Indiana	5,069	88.8	(86.4–91.3)	4,438	64.4	(62.5–66.3)	−3.6
Iowa	2,322	80.3	(77.0–83.6)	1,999	60.4	(57.7–63.1)	−2.8
Kansas	1,797	72.6	(69.2–75.9)	1,521	51.6	(49.0–54.3)	−3.6
Kentucky	3,998	100.7	(97.6–103.9)	3,721	77.5	(74.9–80.0)	−2.8
Louisiana	4,575	111.6	(108.3–114.8)	4,167	87.8	(85.1–90.5)	−2.5
Maine	952	69.7	(65.2–74.1)	743	44.5	(41.3–47.8)	−5.0
Maryland	4,549	92.1	(89.5–94.8)	4,018	65.1	(63.0–67.1)	−3.4
Massachusetts	3,944	65.0	(63.0–67.1)	3,109	43.9	(42.3–45.4)	−4.2
Michigan	8,770	94.0	(92.0–96.0)	7,860	71.3	(69.7–72.9)	−3.1
Minnesota	2,546	57.7	(55.4–59.9)	2,012	36.3	(34.7–37.9)	−4.6
Mississippi	3,307	124.9	(120.6–129.1)	2,974	95.0	(91.5–98.4)	−2.9
Missouri	5,150	93.3	(90.8–95.9)	4,784	72.4	(70.3–74.4)	−2.7
Montana	570	62.1	(57.0–67.2)	623	53.1	(48.9–57.4)	−2.3
Nebraska	968	60.4	(56.6–64.2)	861	46.0	(42.9–49.1)	−3.3
Nevada	1,900	93.1	(88.9–97.3)	1,811	61.5	(58.7–64.4)	−4.0
New Hampshire	884	74.9	(69.9–79.8)	654	42.9	(39.5–46.2)	−6.1
New Jersey	6,321	77.1	(75.2–79.0)	4,933	52.1	(50.6–53.5)	−4.6
New Mexico	1,171	67.8	(63.9–71.7)	1,196	52.1	(49.2–55.1)	−2.8
New York	16,363	89.8	(88.4–91.1)	12,881	62.1	(61.0–63.2)	−3.8
North Carolina	7,443	95.0	(92.9–97.2)	6,730	64.7	(63.2–66.3)	−4.1
North Dakota	478	77.0	(70.1–83.9)	383	53.2	(47.8–58.7)	−4.4
Ohio	10,512	94.8	(93.0–96.6)	8,891	69.1	(67.7–70.6)	−3.5
Oklahoma	3,573	104.9	(101.5–108.4)	3,641	89.8	(86.9–92.8)	−2.6
Oregon	2,227	68.0	(65.1–70.8)	1,888	43.3	(41.4–45.3)	−4.8
Pennsylvania	10,664	82.7	(81.1–84.3)	8,417	58.0	(56.8–59.3)	−3.8
Rhode Island	830	82.0	(76.4–87.6)	597	52.3	(48.1–56.6)	−4.7
South Carolina	3,959	99.8	(96.7–102.9)	3,923	73.8	(71.5–76.2)	−3.5
South Dakota	527	72.9	(66.6–79.1)	468	53.1	(48.2–57.9)	−3.1
Tennessee	6,342	112.7	(109.9–115.4)	6,311	88.8	(86.6–91.0)	−2.9
Texas	16,477	94.3	(92.9–95.8)	15,241	64.4	(63.4–65.4)	−4.3
Utah	846	54.0	(50.3–57.6)	806	36.9	(34.4–39.5)	−3.3
Vermont	396	65.8	(59.3–72.3)	364	47.8	(42.8–52.8)	−3.8
Virginia	5,350	80.4	(78.2–82.6)	4,663	54.6	(53.0–56.2)	−4.2
Washington	3,796	72.7	(70.3–75.0)	3,400	47.1	(45.5–48.7)	−4.5
West Virginia	2,044	101.8	(97.4–106.3)	1,716	74.5	(70.9–78.1)	−3.6
Wisconsin	3,842	75.6	(73.2–78.0)	3,232	52.5	(50.7–54.3)	−3.8
Wyoming	316	66.2	(58.8–73.5)	322	52.8	(46.9–58.7)	−1.6

**Abbreviation:** CI = confidence interval.

* Avoidable deaths from heart disease, stroke, and hypertensive disease are defined as all deaths occurring in persons aged <75 years with an underlying cause of ischemic heart disease, cerebrovascular disease, hypertensive disease, or chronic rheumatic heart disease.

† Per 100,000 population. Rates are age-standardized to the U.S. standard 2000 population.

§ All annual average percentage changes are statistically different from zero at alpha = 0.05.

## Footnotes

* Persons of Hispanic ethnicity might be of any race or combination of races. Persons identified as any of the other racial/ethnic categories were non-Hispanic.

† *West*: Alaska, Arizona, California, Colorado, Hawaii, Idaho, Montana, Nevada, New Mexico, Oregon, Utah, Washington, and Wyoming. *Midwest*: Illinois, Indiana, Iowa, Kansas, Michigan, Minnesota, Missouri, Nebraska, North Dakota, Ohio, South Dakota, and Wisconsin. *Northeast*: Connecticut, Maine, Massachusetts, New Hampshire, New Jersey, New York, Pennsylvania, Rhode Island, and Vermont. *South*: Alabama, Arkansas, Delaware, District of Columbia, Florida, Georgia, Kentucky, Louisiana, Maryland, Mississippi, Oklahoma, North Carolina, South Carolina, Tennessee, Texas, Virginia, and West Virginia.
